# Digital Motor Profiling in Parkinson’s Disease: Long-Term Monitoring Through Wearable Sensors

**DOI:** 10.3390/brainsci16101074

**Published:** 2026-10-07

**Authors:** Alessandro Zampogna, Luigi Borzì, Domiziana Rinaldi, Gabriele Imbalzano, Martina Patera, Marco Falletti, Carlo Alberto Artusi, Caterina Pauletti, Edoardo Bianchini, Leonardo Lopiano, Gabriella Olmo, Antonio Suppa

**Affiliations:** 1 Department of Human Neurosciences, Sapienza University of Rome, 00185 Rome, Italy; alessandro.zampogna@uniroma1.it (A.Z.); martina.patera@uniroma1.it (M.P.); marco.falletti@uniroma1.it (M.F.); caterina.pauletti@uniroma1.it (C.P.); 2 Department of Control and Computer Engineering, Politecnico di Torino, 10129 Turin, Italy; luigi.borzi@polito.it (L.B.); gabriella.olmo@polito.it (G.O.); 3 Department of Neuroscience, Mental Health and Sense Organs (NESMOS), Sapienza University of Rome, 00189 Rome, Italy; domiziana.rinaldi@uniroma1.it; 4 Department of Neuroscience “Rita Levi Montalcini”, University of Turin, 10126 Turin, Italy; 5 Department of Neurosciences, Biomedicine, and Movement, University of Verona, 37124 Verona, Italy; caartusi@gmail.com; 6 Fondazione Policlinico Universitario Campus Bio-Medico, 00128 Rome, Italy; e.bianchini@policlinicocampus.it; 7 Université Grenoble Alpes, CNRS, Grenoble INP, LIG Sangria, 38400 Grenoble, France; 8 IRCCS Neuromed Institute, 86077 Pozzilli, Italy

**Keywords:** Parkinson’s disease, wearable sensors, gait, long-term monitoring, digital motor profiling

## Abstract

**Background/Objectives:** Parkinson’s disease (PD) is clinically heterogeneous, and motor complications are often inadequately captured during routine outpatient assessments. In this cross-sectional observational study, we investigated whether prolonged home-based wearable monitoring could enable digital motor profiling by identifying clinically meaningful patient subgroups through an unsupervised analytical approach. **Methods:** Seventy-six patients with PD underwent standardized clinical evaluation followed by prolonged home-based monitoring using a single waist-worn inertial sensor. Wearable-derived measures of gait, mobility, and complex motor manifestations, including dyskinesia and freezing of gait (FOG), were analyzed using an unsupervised clustering approach based exclusively on sensor-derived variables. The resulting subgroups were subsequently characterized using clinical and digital outcomes, and cluster stability and interpretability were examined through bootstrap and complementary machine-learning analyses. **Results:** Unsupervised clustering identified two distinct digital motor profiles that differed primarily in the burden of motor complications rather than overall disease severity. Although disease duration, Hoehn and Yahr stage, and clinical motor phenotype did not differ significantly, patients in one subgroup exhibited significantly greater dyskinesia, motor fluctuations, and dopaminergic treatment requirements, accompanied by a distinct sensor-derived motor profile during daily life. Dyskinesia-related metrics emerged as the strongest contributors to profile discrimination, and the identified clusters were reproduced with high cross-validated agreement by the surrogate classification model. **Conclusions:** Prolonged home-based wearable monitoring may support clinically meaningful digital motor profiling in PD. Beyond conventional clinical staging, this approach can identify patients with different burdens of motor complications under real-world conditions. Following appropriate validation, objective digital biomarkers could support patient stratification, longitudinal monitoring, and treatment optimization.

## 1. Introduction

Parkinson’s disease (PD) is the second most common neurodegenerative disorder worldwide and is characterized by progressive motor and non-motor manifestations that increasingly compromise functional independence and quality of life [1]. The cardinal motor syndrome is defined by bradykinesia, rigidity, resting tremor, and, in more advanced stages, postural instability [1,2]. L-Dopa remains the most effective symptomatic treatment, but its therapeutic efficacy evolves as the disease progresses. During the early stages of PD, residual nigrostriatal buffering together with the long-duration response preserves stable motor control despite intermittent dosing. As neurodegeneration advances, motor function becomes progressively reliant on the short-duration response, leading to the emergence of motor fluctuations and dyskinesias [3]. These complications represent the main indication for advanced therapies, including continuous dopaminergic drug delivery and deep brain stimulation, underscoring the importance of their early identification to guide personalized treatment strategies [4,5]. Assessing motor complications remains challenging because their expression varies with medication timing, emotional state, and environmental context [6]. Brief outpatient evaluations often fail to capture real-world motor function, while therapeutic decisions largely rely on patient and caregiver interviews or diaries, which are prone to recall bias, subjective interpretation, cognitive impairment, and poor adherence. These limitations have driven increasing interest in wearable-derived digital biomarkers [7,8].

Wearable inertial sensors enable objective, quantitative, and continuous assessment of motor performance during daily life and have demonstrated good accuracy in measuring motor impairment in PD [7,9,10]. Despite growing evidence supporting their analytical accuracy [11,12], most studies have focused either on validating individual digital biomarkers or quantifying specific motor manifestations during laboratory assessments and long-term home monitoring [13,14,15,16]. Specifically, evidence from long-term home-based monitoring has been largely limited to demonstrating the ability of wearable sensors to objectively quantify individual motor domains, such as gait characteristics, motor fluctuations, dyskinesia, and freezing of gait (FOG), and to correlate these digital measures with conventional clinical scales obtained during episodic outpatient evaluations [13,14,15,16]. To our knowledge, no previous study has applied an unsupervised clustering approach to long-term home-based wearable-derived motor data to stratify PD patients and identify distinct digital motor profiles. Given the marked heterogeneity of PD, data-driven digital profiling may reveal complementary dimensions of motor dysfunction beyond conventional clinical assessment, which would help improve patient assessment and therapeutic management. Among available digital biomarkers, gait is particularly well suited for this purpose because it integrates locomotor, postural, sensorimotor, and executive functions that progressively deteriorate during the disease [17,18,19]. Moreover, gait can be monitored continuously using a single wearable sensor, providing a simple, scalable, and low-burden approach for home-based assessments [14].

In this cross-sectional observational study, we investigated whether prolonged home-based motor monitoring with a single wearable sensor could enable objective digital motor profiling in a heterogeneous cohort of patients with PD. We applied a PCA-based unsupervised clustering approach exclusively to spatiotemporal gait parameters and other sensor-derived measures collected during daily life, without incorporating any clinical information, to determine whether distinct digital motor profiles could be identified using wearable-derived motor data alone. We subsequently evaluated whether the resulting wearable-derived profiles differed with respect to disease severity, motor complications, conventional clinical measures, and sensor-derived mobility metrics. We hypothesized that prolonged home-based monitoring would identify clinically meaningful profiles reflecting different burdens of motor complications, thereby supporting the use of wearable-derived digital biomarkers as practical tools for patient stratification and therapeutic decision-making.

## 2. Materials and Methods

### 2.1. Study Design

This multicenter cross-sectional observational study was conducted in accordance with the Strengthening the Reporting of Observational Studies in Epidemiology (STROBE) guidelines (Supplementary Material S1) [20]. The study protocol was approved by the Institutional Review Board of Sapienza University of Rome, Italy (Prot. 0372/2022), on 10 May 2022, and written informed consent was obtained from all participants before enrollment. All study procedures were carried out in accordance with the ethical principles of the Declaration of Helsinki.

### 2.2. Participants

Patients were consecutively recruited from the Movement Disorders outpatient clinics of Sapienza University of Rome and the University of Turin between January 2023 and September 2025. Eligible participants had a diagnosis of idiopathic PD established according to current international diagnostic criteria [21], were receiving chronic dopaminergic therapy, and were able to walk independently (Hoehn and Yahr—H&Y stage ≤ 4). Exclusion criteria included possible or probable atypical parkinsonism, previous ablative functional neurosurgery, severe cognitive impairment preventing adherence to study procedures, inability to walk independently, and neurological, orthopedic, or rheumatological disorders that could affect gait performance. Patients receiving medications expected to substantially interfere with gait assessment during the monitoring period were also excluded. The levodopa equivalent daily dose (LEDD) was calculated using standardized conversion formulas [22].

The cohort included in the present study has been described previously in studies addressing different research questions [23,24]. Specifically, the first study focused on the objective quantification and clinical correlates of dyskinesia and FOG during daily life [23], whereas the second compared real-world mobility and motor fluctuations between patients receiving L-Dopa/Carbidopa intestinal gel and those receiving conventional oral therapy [24]. In contrast, the present study analyzed the complete multidimensional wearable dataset using an unsupervised, data-driven approach to identify digital motor profiles independently of predefined clinical groups or specific motor manifestations. Accordingly, although participant recruitment, clinical evaluation, and home-based wearable monitoring followed the same standardized protocols, the present study addresses a distinct research question using a different analytical approach. 

### 2.3. Clinical Assessment

Before home monitoring, all participants underwent a standardized neurological evaluation performed by movement disorder specialists. Clinical assessment included the H&Y staging scale, the Movement Disorder Society–Unified Parkinson’s Disease Rating Scale (MDS-UPDRS) part I-II-III-IV, the Unified Dyskinesia Rating Scale (UDysRS parts III–IV), the Wearing-Off Questionnaire-19 (WOQ-19), the Freezing of Gait Questionnaire (FOG-Q), the Parkinson’s Disease Questionnaire-39 (PDQ-39), the Parkinson’s Disease Sleep Scale (PDSS), the Montreal Cognitive Assessment (MoCA), the Frontal Assessment Battery (FAB), and the Beck Depression Inventory-II (BDI-II).

To better reflect patients’ habitual motor performance during everyday activities, all evaluations were performed in the ON medication state, approximately one hour after oral levodopa administration or, in patients receiving levodopa/carbidopa intestinal gel infusion, after stable therapeutic infusion had been achieved.

### 2.4. Home-Based Monitoring Protocol

Immediately after the clinical evaluation, participants underwent prolonged motor monitoring during their usual daily activities using a single wearable inertial sensor (STAT-ON™, Sense4Care, Barcelona, Spain) [25]. Participants were instructed to wear the device on the left side of the waist using an elastic belt for at least four consecutive days and a minimum of eight hours per day during waking hours. The device was removed only during sleep or for activities incompatible with its use, such as water-based activities. Caregivers were permitted to assist with sensor placement whenever necessary.

Before delivery, trained study personnel configured the device using participant-specific clinical information, including age, H&Y stage, and lower-limb length measured from the left anterior superior iliac spine to the floor. These parameters were used to calibrate the device algorithms and improve the accuracy of gait analysis and activity detection. Once correctly positioned, the sensor operated autonomously throughout the monitoring period without requiring manual activation, calibration, or participant interaction. At the end of the recording period, participants returned the device to the outpatient clinic, where adherence was verified, and the recorded data were downloaded for offline analysis.

### 2.5. Wearable Device and Embedded Signal Processing

Motor performance was continuously monitored using the CE-certified Class IIa wearable medical device STAT-ON™ (9 × 6.3 × 2.1 cm, 86 g), specifically designed for long-term assessment of motor manifestations in PD under unrestricted daily-life conditions [25]. The device integrates two ultra-low-power triaxial accelerometers, two microcontrollers, onboard memory, and Bluetooth Low Energy connectivity [26]. Acceleration signals are acquired at 50 Hz with a ±6 g measurement range and 12-bit resolution, enabling up to seven consecutive days of continuous recording.

Signal processing was performed directly on the device using previously validated embedded machine-learning algorithms [27,28,29,30]. Raw acceleration signals were segmented into consecutive 3.2-s windows with 50% overlap, and walking periods were identified through a support vector machine classifier based on temporal- and frequency-domain features. Individual strides were subsequently detected from the forward acceleration component, while the first and last strides of each walking bout were excluded to improve measurement consistency. The embedded algorithms automatically estimated step length, stride speed, cadence, stride fluidity (reflecting stride-to-stride smoothness and regularity), and total step count, with gait-related outcomes summarized over 1-min epochs. Dedicated validated algorithms simultaneously detected dyskinesias and FOG and estimated the proportion of time spent in the ON, OFF, and Intermediate motor states throughout the monitoring period. Dyskinesia measures were calculated over consecutive 10-min intervals, whereas FOG episodes and their duration were quantified on a 1-min basis. All recordings were stored in the device memory and subsequently transferred to the manufacturer’s software platform for analysis. Details of the hardware architecture, algorithm development, and technical validation have been reported elsewhere [27,28,29,30]. For the present study, the analyses were restricted to spatiotemporal gait variables and the additional instrumental measures generated by the embedded processing algorithms.

### 2.6. Data Preprocessing

Data were preprocessed before statistical analysis to maximize the reliability of gait estimates. STAT-ON-derived features were aggregated at the subject level. For spatiotemporal gait parameters (stride fluidity, step length, stride speed, and cadence), the mean and coefficient of variation (CV) were calculated to characterize average performance and intra-subject variability. For discrete behavioral events and cumulative measures (gait bouts, steps, FOG episodes, falls, postural transitions, movement amount) and device-state annotations (ON/OFF state and dyskinesia), total counts or cumulative durations were calculated over the monitoring period. The resulting subject-level feature matrix did not include any missing value.

Principal component analysis (PCA) was applied to reduce dimensionality while preserving most of the variance in the dataset. The minimum number of principal components explaining at least 90% of the cumulative variance was retained, and the resulting PCA-transformed dataset was used for all subsequent clustering analyses.

### 2.7. Outcome Measures

The primary outcome measures included spatiotemporal gait parameters (stride fluidity, step length, stride speed, and cadence) and additional wearable-derived motor measures obtained during long-term monitoring, including gait bouts, steps, FOG episodes, falls, postural transitions, movement amount, ON/OFF state estimates, and dyskinesia measures. Dyskinesia severity was assessed using Mean Dyskinesia Power, reflecting dyskinesia intensity; Normalized Dyskinesia Duration, reflecting dyskinesia duration; and Dyskinesia Continuity, reflecting the temporal persistence of dyskinetic episodes. These wearable-derived measures constituted the feature set used for the unsupervised clustering analysis.

Secondary outcome measures included clinical and demographic characteristics, disease severity, motor complications, cognitive and neuropsychiatric measures, and conventional clinical scale scores, which were used to characterize and compare the sensor-derived clusters.

### 2.8. Clustering Procedures and Statistical Analysis

Continuous variables are summarized as mean ± standard deviation or median (interquartile range [IQR]) according to data distribution, whereas categorical variables are reported as frequencies and percentages. Normality was assessed using the Shapiro–Wilk test.

Unsupervised clustering was performed using the K-means algorithm applied to the PCA-transformed feature space. The optimal number of clusters was determined by evaluating candidate k values from 2 to 14 using a composite internal validation score combining the normalized Silhouette coefficient and Calinski–Harabasz index. The final model was fitted using the selected k with 100 random initializations to improve centroid stability. Cluster stability was assessed using bootstrap resampling (200 iterations). In each iteration, subjects were resampled with replacement, and the complete clustering procedure was repeated. The resulting bootstrap cluster assignments were compared with the corresponding reference cluster assignments from the original dataset using the Adjusted Rand Index (ARI) and Normalized Mutual Information (NMI). ARI quantified the agreement between the two partitions while correcting for agreement expected by chance, whereas NMI quantified the shared information between the bootstrap and reference cluster assignments. Higher ARI and NMI values indicated greater cluster stability. Moreover, to assess the robustness of the clustering solution to dimensionality reduction and the contribution of individual sensor-derived motor domains, additional sensitivity analyses were performed using both leave-one-domain-out and domain-only approaches. A detailed description of the clustering procedure, internal validation metrics, mathematical formulations, and robustness and sensitivity analyses is provided in the Supplementary Materials S2.

Clinical variables were not included in the clustering procedure and were used exclusively for post hoc characterization of the identified clusters. Specifically, clinical and instrumental characteristics were compared between clusters to identify variables associated with cluster membership. Normally distributed continuous variables were analyzed using independent two-sample *t*-tests, whereas non-normally distributed variables were compared using the Mann–Whitney U test. Effect sizes were calculated as Cohen’s d for parametric comparisons and rank-biserial correlation for non-parametric analyses. Categorical variables were compared using the χ^2^ test or Fisher’s exact test when expected cell frequencies were below five, with Cramér’s V used to quantify effect size.

A two-sided *p* < 0.05 was considered statistically significant. To account for multiple comparisons across clinical variables, false discovery rate (FDR) correction was applied using the Benjamini–Hochberg procedure, with adjusted *p* < 0.05 considered statistically significant.

### 2.9. Classification and Explainability

A supervised machine-learning approach based on a Random Forest classifier was used to distinguish the identified clusters. The random forest analysis was intended to assess cluster discriminability and feature contributions rather than to provide independent validation of the clustering solution. Model performance and interpretability were evaluated using 5-fold stratified cross-validation, with class distributions preserved across all folds. The dataset was randomly shuffled using a fixed seed to ensure reproducibility. Within each fold, 80% of the data were allocated to model training and the remaining 20% to testing. The random forest classifier was trained using 100 trees, square-root feature sampling, balanced class weights, and a fold-specific random seed. Parallel computation was enabled throughout the training procedure. Model performance on the held-out test sets was evaluated using accuracy, balanced accuracy, and F1-score, with results reported as mean ± standard deviation across the five folds. Model interpretability was investigated using SHAP (SHapley Additive exPlanations) values computed with TreeExplainer. To avoid information leakage, SHAP values were calculated exclusively on the test set of each fold and subsequently concatenated across folds to generate a cross-validated explanation matrix. Global feature importance was quantified as the mean absolute SHAP value for each feature, reflecting its average contribution to model predictions irrespective of direction, and features were ranked accordingly.

All analyses were performed in Python (version 3.11) using NumPy (version 1.26.4), Pandas (version 3.0.5), SciPy (version 1.17.0), Matplotlib (version 3.10.8), scikit-learn (version 1.8.0), and SHAP (version 0.45.0).

**Figure 1:**
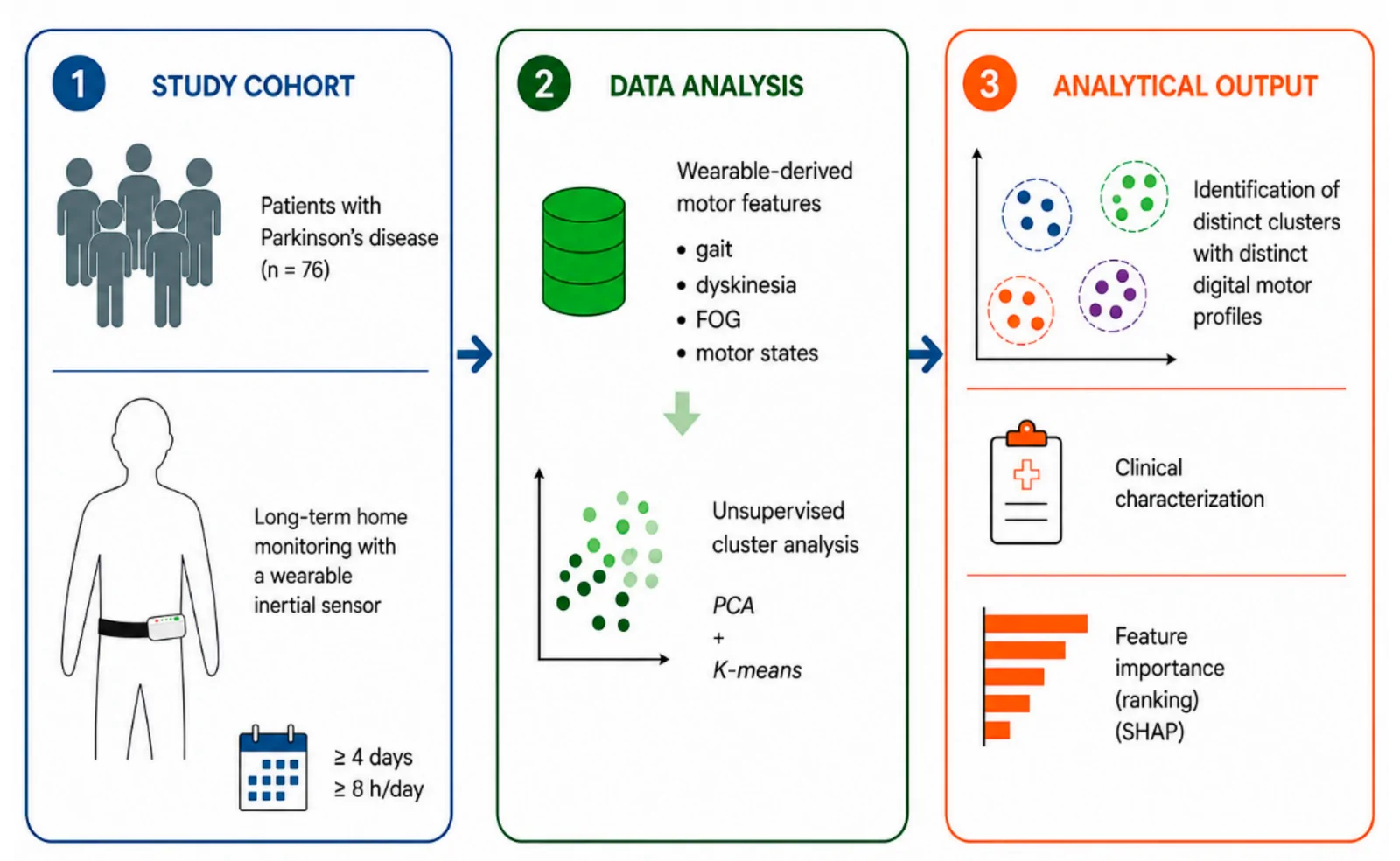
Schematic overview of the study design. Patients with Parkinson’s disease underwent long-term home monitoring using a single wearable inertial sensor during free-living daily activities. Wearable-derived motor features were used for unsupervised clustering to identify distinct digital motor profiles, which were subsequently characterized using clinical comparisons and SHAP-based feature importance analysis. The depicted clusters are illustrative and do not represent the number or separation of the profiles identified in the study.

A schematic overview of the study design and analytical workflow is shown in Figure 1.

## 3. Results

### 3.1. Study Cohort and Home-Based Monitoring

A total of 76 PD patients were enrolled according to the study eligibility criteria. Participants agreed to undergo the experimental procedures and provided written informed consent before study participation. All enrolled patients successfully completed the study protocol without dropouts or procedure-related issues. The mean duration of home-based monitoring was 6.7 ± 1.3 days (median: 6 days, IQR: 6–8 days). The study cohort was clinically heterogeneous, including patients across different stages of disease severity. The demographic and clinical characteristics of the study population are summarized in Table 1.

### 3.2. Dimensionality Reduction and Clustering

**Figure 2:**
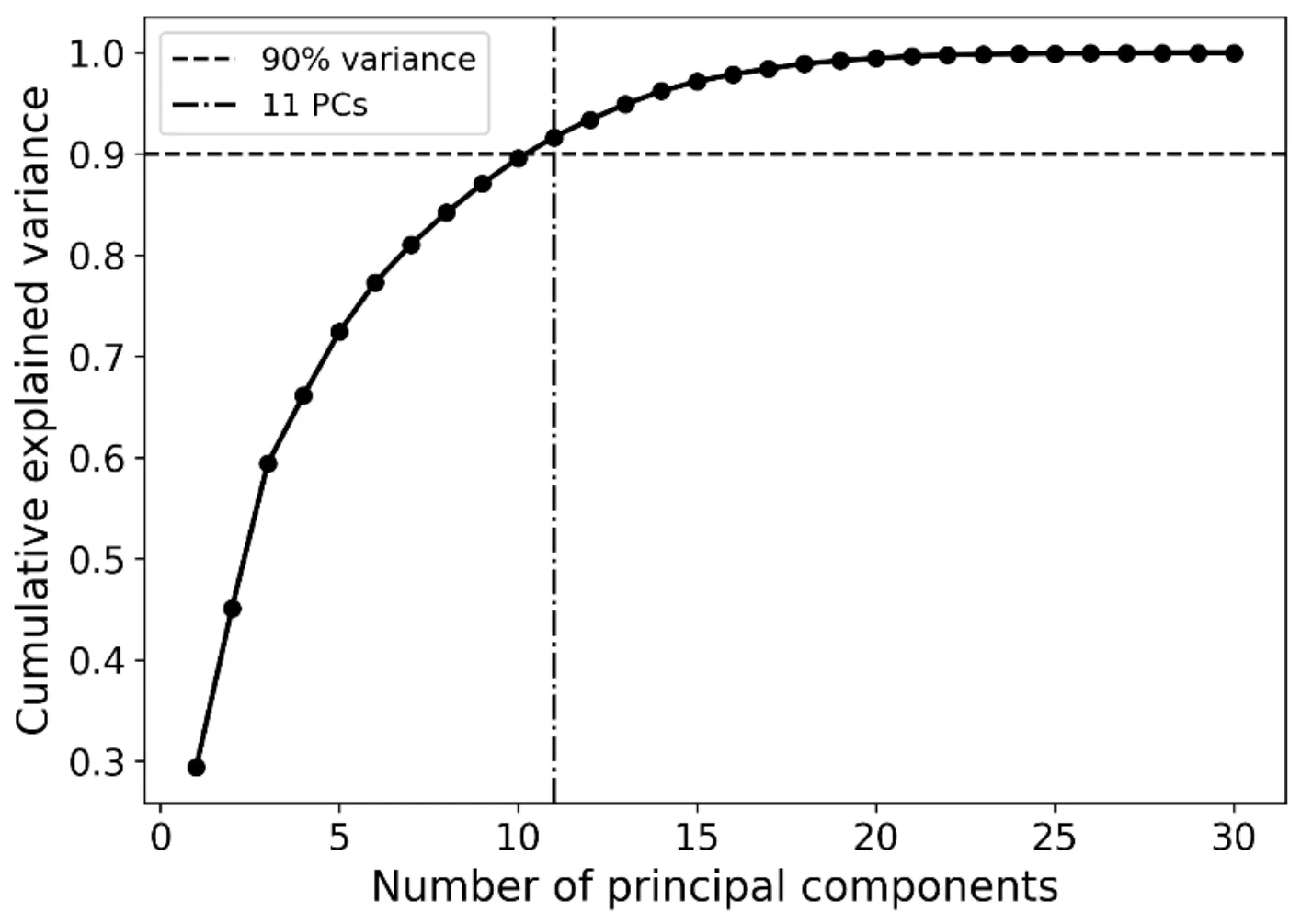
Cumulative explained variance as a function of the number of retained principal components.

Principal component analysis retained 11 principal components, explaining 91.7% of the cumulative variance (Figure 2).

**Figure 3:**
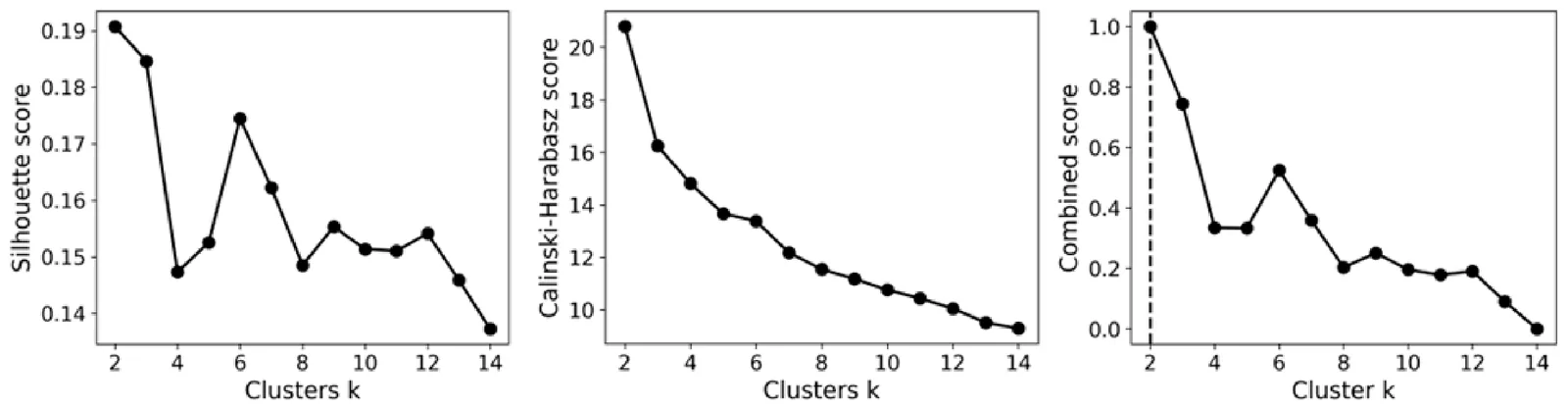
Silhouette score, Calinski–Harabasz index, and composite score across candidate clustering solutions (k = 2–14).

The optimal clustering solution was identified by evaluating candidate k values from 2 to 14 using the Silhouette score, Calinski–Harabasz (CH) index, and their composite score (Figure 3). Silhouette values were consistently low across all candidate solutions (range: 0.137–0.191), indicating modest separation between clusters in the reduced feature space. The highest Silhouette score was observed for the two-cluster solution (≈0.191), with a secondary local increase around *k* = 6, though values generally declined thereafter. The CH index likewise reached its maximum for the two-cluster solution (≈20.8) and decreased monotonically with increasing *k*. Integration of both metrics into a composite score again identified the two-cluster solution as optimal (≈1.00), with substantially lower scores for all subsequent solutions. Accordingly, the two-cluster solution was selected for all subsequent analyses.

**Figure 4:**
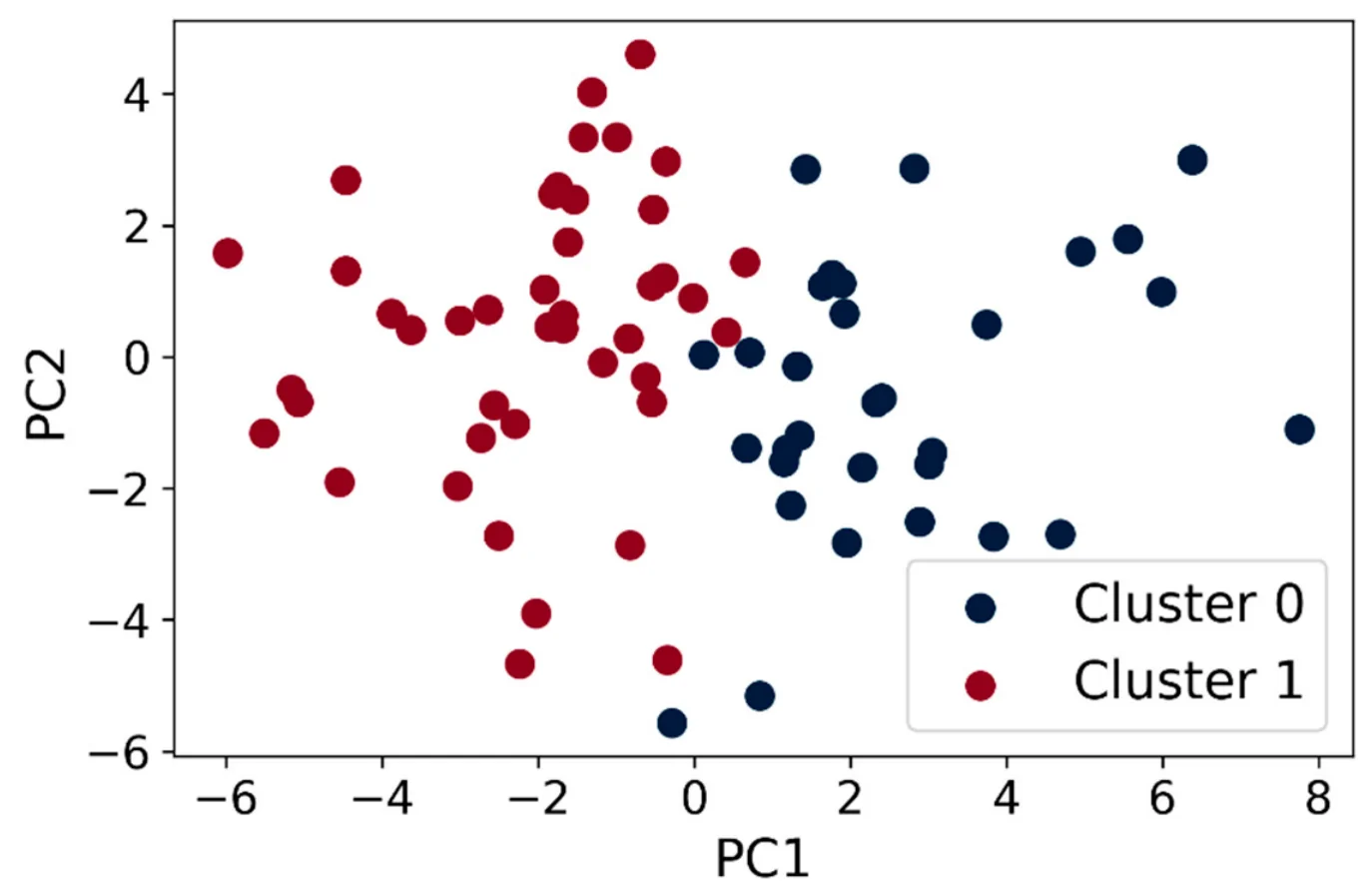
Two-dimensional visualization of the clustering solution based on the first two principal components (PC1 and PC2). Cluster 0 = High Motor Complication (HMC); Cluster 1 = Low Motor Complication (LMC).

Projection of the clustering solution onto the first two principal components (Figure 4) showed that cluster separation occurred predominantly along the first principal component (PC1), whereas considerable overlap was observed along the second principal component (PC2). This pattern indicates that discrimination between clusters was primarily driven by variation captured by PC1, while the overlap along PC2 suggests that cluster membership reflected the combined contribution of multiple variables rather than a simple two-dimensional structure.

Bootstrap resampling demonstrated moderate-to-high clustering stability, with a mean ARI of 0.79 ± 0.17 (median, 0.85; IQR, 0.71–0.95; range, 0.10 to 1.00) and mean NMI of 0.76 ± 0.17 (median, 0.78; IQR, 0.65–0.91; range, 0.16 to 1.00) across 200 iterations. Although a subset of bootstrap samples yielded lower agreement, most iterations showed substantial concordance with the reference clustering.

Sensitivity analyses further confirmed that the clustering solution was robust to dimensionality reduction and indicated that dyskinesia-related features contributed most strongly to the identified cluster structure (see Supplementary Materials S2 for detailed results of sensitivity analyses).

### 3.3. Clinical and Digital Profiling of the Identified Clusters

After correction for multiple comparisons, the two clusters differed significantly in dopaminergic treatment requirements and motor complications. Compared with Cluster 1, patients in Cluster 0 exhibited a greater burden of motor complications, as reflected by higher MDS-UPDRS Part IV, UDysRS Part III and IV, and WOQ-19 scores, together with higher LEDDs. Conversely, no significant between-cluster differences were observed in age, disease duration, age at disease onset, H&Y stage, global motor severity (MDS-UPDRS Part III), activities of daily living (MDS-UPDRS Part II), non-motor symptoms (MDS-UPDRS Part I), gait impairment (FOG-Q), cognition (MoCA and FAB), mood (BDI-II), sleep quality (PDSS), quality of life (PDQ-39), or conventional motor phenotype after correction for multiple comparisons. Accordingly, based on their clinical profiles, Cluster 0 was designated as the High Motor Complication (HMC) cluster and Cluster 1 as the Low Motor Complication (LMC) cluster. Clinical and demographic comparisons between the two clusters are summarized in Table 2.

Comparison of sensor-derived outcomes revealed distinct motor profiles between the HMC and LMC clusters during daily-life monitoring. Compared with the LMC cluster, patients in the HMC cluster exhibited lower cadence together with higher stride fluidity and stride fluidity variability. They also experienced a greater burden of FOG, reflected by a higher number of episodes and longer FOG duration. In addition, wearable-derived measures indicated a greater burden of dyskinesia in the HMC cluster, with both higher average dyskinesia intensity (Mean Dyskinesia Power) and longer normalized dyskinesia duration. Finally, the HMC cluster showed greater overall daily movement amount and postural transitions. No significant between-cluster differences were observed for step length, stride speed, or the normalized number of walking bouts. Sensor-derived outcomes for the LMC and HMC clusters are summarized in Table 3.

### 3.4. Surrogate Classification and Model Explainability

The random forest achieved a mean accuracy of 93.5%, a mean balanced accuracy of 93.5%, and a mean F1-score of 94.3% across the five cross-validation folds (Table 4), indicating that the cluster assignments derived from the unsupervised analysis could be reproduced with high accuracy from the input features. Classification performance remained consistently high across folds.

**Figure 5:**
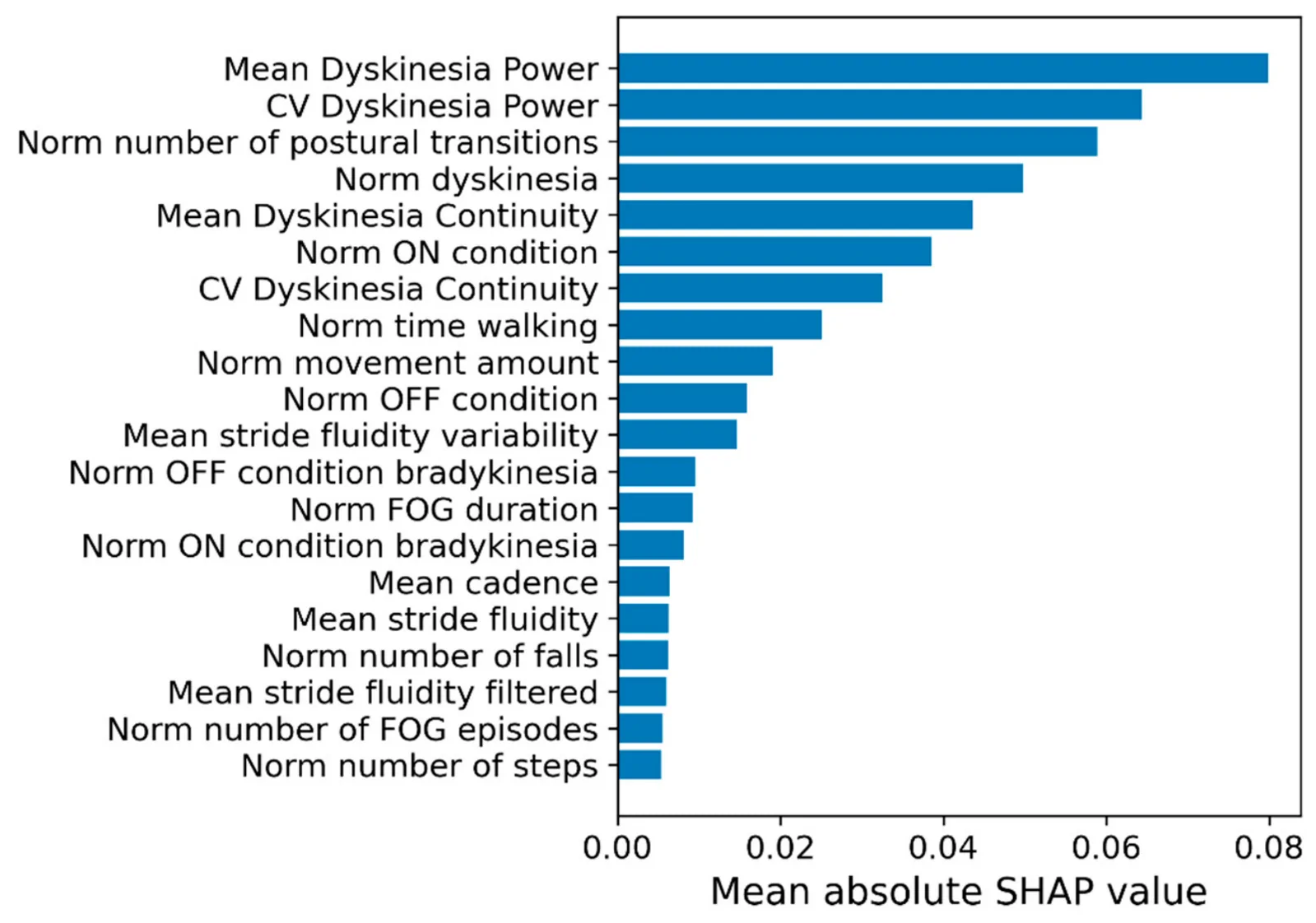
SHAP-based feature importance for reproducing the PCA-derived cluster assignments. Features are ranked according to their mean absolute SHAP values, indicating their average contribution to the random forest surrogate model predictions across all samples. Higher mean absolute SHAP values denote greater importance for cluster classification. SHAP, Shapley Additive Explanations; CV, coefficient of variation; FOG, freezing of gait; Norm, normalized to valid monitoring (wear) time.

Feature importance analysis based on SHAP values identified the mean and coefficient of variation (CV) of Dyskinesia Power as the strongest contributors to cluster classification, followed by the normalized number of postural transitions, normalized dyskinesia duration, and the mean Dyskinesia Continuity score (Figure 5). Measures reflecting overall mobility and physical activity, including normalized walking time and normalized movement amount, also ranked among the most influential features, whereas gait-related measures, FOG metrics, and step count contributed comparatively less to the model.

**Figure 6:**
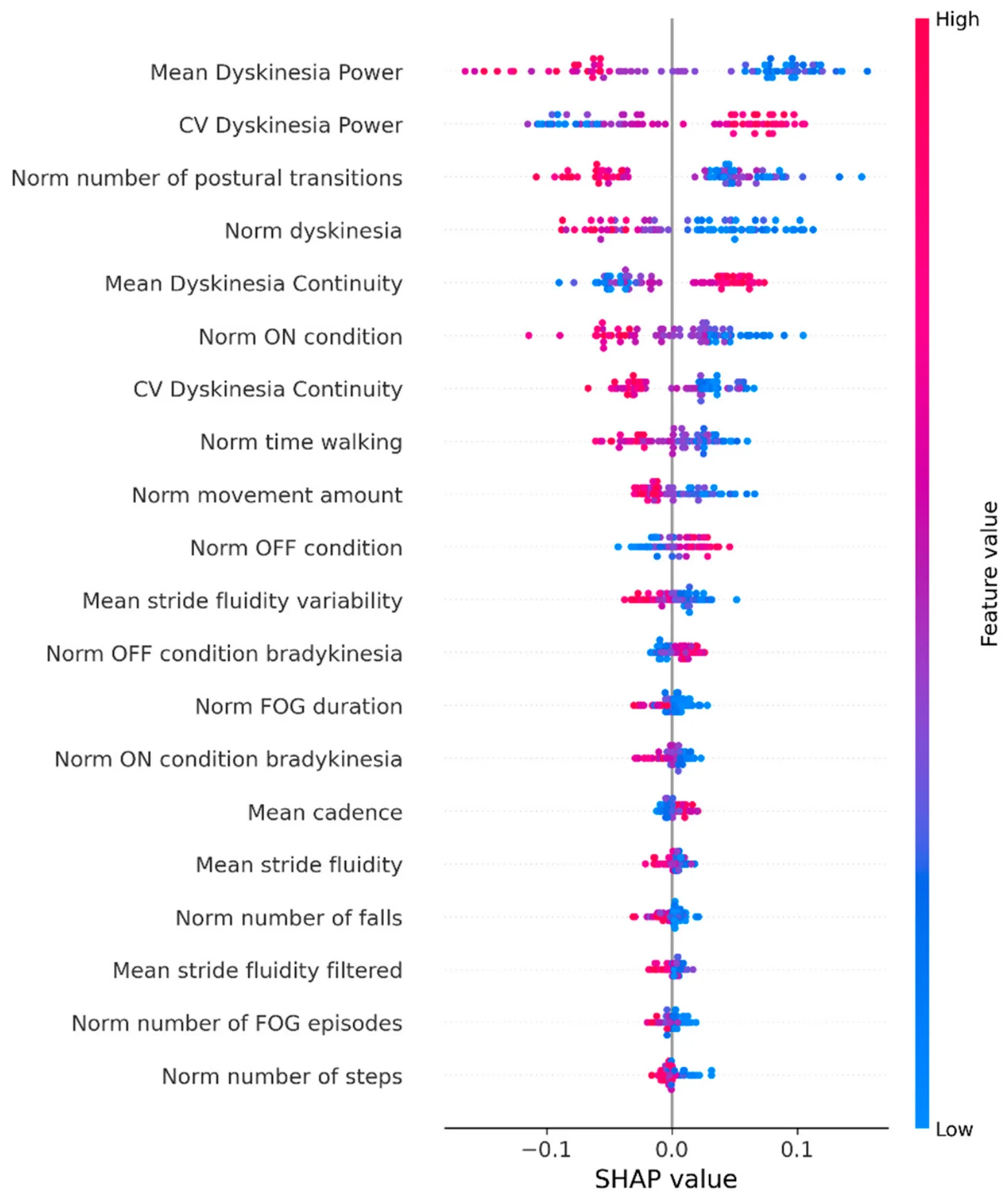
SHAP summary plot of feature importance for cluster classification. SHAP (Shapley Additive Explanations) summary plot showing the contribution of wearable-derived features to the random forest classification of the identified clusters. Features are ranked according to their overall importance. Each dot represents one participant, with color indicating the feature value (blue = low, red = high). Negative SHAP values indicate greater contribution to classification into the HMC cluster, whereas positive SHAP values indicate greater contribution to classification into the LMC cluster. SHAP, Shapley Additive Explanations; CV, coefficient of variation; FOG, freezing of gait; Norm, normalized to valid monitoring (wear) time.

The SHAP summary plot further illustrated the direction of these associations (Figure 6). Higher values of Mean Dyskinesia Power, normalized dyskinesia duration (Norm dyskinesia), normalized movement amount (Norm movement amount), normalized walking time (Norm time walking), normalized number of postural transitions (Norm number of postural transitions), and CV of the Dyskinesia Continuity score contributed predominantly toward classification into the HMC cluster. Conversely, higher values of the Mean Dyskinesia Continuity score and normalized OFF condition (Norm off condition) favored classification into the LMC cluster.

## 4. Discussion

By exclusively leveraging wearable-derived data collected during long-term home-based motor monitoring, our unsupervised clustering analysis identified two clinically meaningful subgroups within a heterogeneous cohort of patients with PD. Although clustering was performed solely on digital motor features, the resulting groups differed primarily in clinical measures of motor complications while also exhibiting distinct sensor-derived motor profiles. Collectively, these findings identified a Low Motor Complication (LMC) cluster of patients with PD, characterized by a lower burden of fluctuations and dyskinesia together with a milder impairment of selected digital gait and mobility measures, and a High Motor Complication (HMC) cluster, characterized by more severe motor complications and greater impairment of sensor-derived motor performance.

To minimize potential confounding in our study, we adopted standardized clinical and home-monitoring procedures for all participants and performed unsupervised clustering exclusively on wearable-derived variables, while demographic and clinical characteristics were reserved for post hoc validation of the identified clusters. Cluster robustness was supported by bootstrap resampling, Random Forest surrogate classification, and SHAP-based explainability analysis, reducing the likelihood that the identified digital profiles reflected random data partitioning rather than reproducible underlying patterns. In addition, free-living monitoring in everyday settings allowed us to capture ecologically valid conditions, avoiding the influence of artificial constraints of in-hospital evaluations.

As the first methodological finding, our unsupervised analytical approach showed good technical performance. PCA retained more than 90% of the original variance while substantially reducing data dimensionality, internal validation metrics consistently supported the selection of a two-cluster solution, and bootstrap resampling demonstrated good clustering stability. Sensitivity analyses further supported the robustness of the clustering solution to dimensionality reduction. Although cluster separation in the reduced feature space was modest, this finding is consistent with the continuous nature of motor impairment in PD, where patients are distributed along a clinical continuum rather than forming sharply distinct categories [31]. Together, these results suggest that long-term wearable-derived motor data contain a reproducible low-dimensional structure. Furthermore, the ability of PCA to preserve more than 90% of the original variance using a limited number of principal components indicates that the high-dimensional sensor data can be effectively compressed, likely because many extracted motor features capture correlated aspects of motor behaviour [32].

### Digital Motor Profiling in Parkinson’s Disease

Our unsupervised, data-driven analysis of prolonged home-based wearable monitoring identified two distinct motor profiles within our cohort of patients with PD. The two clusters differed primarily in the clinical burden of motor complications, with the HMC group exhibiting more severe dyskinesias and motor fluctuations than the LMC group. These differences were accompanied by higher dopaminergic medication requirements in the HMC group, likely reflecting greater therapeutic efforts to manage a more compromised motor condition. Disease duration, H&Y stage, and clinical motor phenotype distribution (tremor-dominant, akinetic-rigid, or mixed) did not differ significantly between clusters after FDR correction. Accordingly, the identified clusters should not be interpreted as simply distinguishing patients with earlier versus more advanced disease or according to the presence of tremor. Moreover, the relatively modest silhouette coefficient (approximately 0.19) indicates substantial overlap between the identified clusters in the multivariate feature space, which therefore should not be interpreted as sharply separated PD phenotypes. In contrast, they represent partially distinct data-driven digital motor profiles within a broader clinical continuum, which primarily reflects differences in the burden of motor complications objectively captured during prolonged real-world monitoring. The finding of substantial differences in motor complication burden in the absence of statistically significant differences in disease duration and clinical stage should not be interpreted as evidence of equivalence or independence from disease severity but is consistent with current concepts of PD progression [31]. Motor complications are not solely determined by disease duration or overall disease stage but arise from the complex interplay between the extent of nigrostriatal degeneration, cumulative dopaminergic exposure, and individual susceptibility [33,34,35]. Consequently, patients with similar disease duration and clinical stage may experience markedly different patterns of motor fluctuations and dyskinesia [34,36]. Our findings suggest that prolonged wearable monitoring may capture this clinically relevant dimension of disease heterogeneity and inter-individual variability, providing information that complements conventional clinical staging. This concept aligns with the broader framework of deep digital profiling, in which multidimensional digital measurements are used to characterize disease heterogeneity beyond conventional clinical classifications [37,38,39]. From a clinical perspective, the ability to identify patients with a greater burden of motor complications from unobtrusively acquired home-based data could provide clinically meaningful insights, particularly when motor fluctuations and dyskinesias are not adequately represented during outpatient visits. Such information could ultimately facilitate patient stratification, longitudinal monitoring, and the identification of individuals who may benefit from treatment reassessment or evaluation for device-aided therapies [40,41].

Beyond distinguishing patients according to the overall burden of motor complications assessed by clinical scales, sensor-derived metrics also provided insights into how these complications influence motor behaviour during everyday life. Indeed, compared with the LMC cluster, patients in the HMC cluster exhibited a characteristic combination of greater FOG burden, altered gait dynamics, increased dyskinesia, and higher levels of daily movement and postural transitions. We hypothesize that this digital motor profile, derived from prolonged home monitoring, captures the dynamic expression of motor complications rather than simply reflecting patients’ overall motor impairment. The greater burden of FOG observed in the HMC cluster is consistent with previous evidence showing that FOG becomes increasingly prevalent in fluctuating patients with reduced dopaminergic buffering capacity [42,43]. This greater objective burden of FOG was not accompanied by significant between-cluster differences in FOG-Q scores. This apparent discrepancy likely reflects the well-recognized challenges of assessing FOG using patient-reported outcomes [44,45]. Because FOG is an episodic and context-dependent phenomenon, patients may fail to recognize individual episodes accurately, underestimate their frequency or severity, or have difficulty recalling events experienced during daily life [46]. These limitations are particularly relevant given that FOG frequently coexists with cognitive impairment, which is known to increase reporting bias [47]. Moreover, day-to-day reliability may vary across digital measures, with greater stability for mean-based measures and lower stability for episodic events such as FOG and falls, further supporting the value of multi-day monitoring for a more reliable characterization of motor behaviour [48]. Collectively, these findings further support the added value of continuous objective monitoring for capturing the real-world burden of FOG. In line with the greater burden of FOG, HMC patients also exhibited altered gait dynamics compared with LMC patients. Specifically, they showed reduced cadence and greater stride fluidity variability despite preserved stride speed and step length. The apparently counterintuitive increase in mean stride fluidity observed in HMC patients should be interpreted together with its greater variability. Indeed, this combination may reflect a less stable gait pattern, with periods of relatively preserved fluidity alternating with greater gait disruption, rather than an overall improvement in gait performance. Nevertheless, a potential influence of device-related measurement artefacts or dyskinesia-related movements on stride fluidity estimates cannot be excluded. Overall, these findings suggest that temporal aspects of gait may be more sensitive to motor complications than conventional spatiotemporal gait parameters. Our findings are consistent with previous reports showing that gait rhythmicity deteriorates in patients with motor fluctuations even when average gait velocity remains relatively preserved through compensatory mechanisms [49,50]. They are also in agreement with previous studies demonstrating greater impairment of gait performance in patients with a higher burden of FOG [23,51,52,53]. From a pathophysiological perspective, these findings support the concept that progressive dysfunction of the basal ganglia affects not only the severity of individual motor symptoms but also the temporal organization and stability of motor behaviour [33,54]. The concomitant increase in daily movement amount and postural transitions likely reflects a more fragmented and variable motor pattern driven by motor fluctuations rather than greater functional mobility. Moreover, it cannot be excluded that the higher daily movement observed in HMC patients was, at least in part, driven by the greater intensity and duration of dyskinesias detected by the wearable sensors. In this regard, our findings further support previous studies demonstrating that wearable sensors are reliable tools for quantifying L-Dopa-induced dyskinesias under real-world conditions, where abnormal involuntary movements fluctuate considerably throughout the day [15,55,56].

A final comment concerns the feature importance analysis, which identified the sensor-derived characteristics that primarily drove the distinction between the two motor profiles. Consistent with the greater burden of motor complications observed in the HMC cluster, dyskinesia-related metrics, particularly the mean and variability of Dyskinesia Power, emerged as the strongest contributors to cluster classification. This finding was further supported by the sensitivity analyses, in which the dyskinesia domain showed the highest agreement with the original clustering solution when considered alone (90.8%), while its exclusion produced the greatest reduction in subject-level cluster agreement (65.8%). Accordingly, the objective measures of dyskinesia captured a substantial proportion of the variability underlying the digital motor profiles identified in our cohort. This finding may be due to the dynamic nature of dyskinesias, which fluctuate markedly throughout the day and are therefore optimally captured by prolonged home-based monitoring [57,58,59]. Measures reflecting overall mobility, such as daily movement amount and postural transitions, also showed substantial discriminative value, suggesting possible changes in patients’ spontaneous motor behaviour during everyday life or reflecting the indirect impact of dyskinesia. Compared with dyskinesia-related metrics, gait-related variables and FOG measures contributed less to the classification model. This finding is consistent with the observation that several conventional locomotor parameters, including stride speed and step length, did not differ significantly between the two clusters. It is also in line with the clinical observation that the main distinction between the identified patient subgroups was driven by motor complications rather than by global ambulatory impairment. Finally, it should be considered that gait- and FOG-related metrics can only be quantified during walking episodes and therefore reflect only a portion of patients’ daily motor behaviour. In contrast, dyskinesia-related measures can be continuously assessed over a much larger proportion of the recording period, including periods in which patients are standing or otherwise not walking, potentially increasing their discriminative value [60].

Overall, our findings support the clinical value of prolonged home-based wearable monitoring as a complementary tool for conventional clinical assessment. While standard clinical scales remain essential for staging disease severity, continuous objective monitoring provides additional information on the real-world expression of motor complications, enabling the identification of patients with a greater burden of motor complications that may not be fully captured during routine outpatient evaluations. This complementary information provides an objective characterization of motor complications in free-living settings, potentially helping clinicians to relate these manifestations to the impact on patients’ daily life. The prominent contribution of dyskinesia-related metrics to cluster discrimination further suggests that the principal added value of wearable technology lies in its ability to objectively quantify the severity, duration, and temporal variability of dyskinesias during everyday life. These measures may therefore provide potential digital biomarkers for future patient stratification, pending prospective clinical validation. When considering this study, some limitations should be considered. First, its cross-sectional design precludes conclusions regarding the temporal evolution of the identified digital motor profiles, warranting longitudinal studies to determine their stability and prognostic value. Second, although the identified clusters were supported by multiple internal validation approaches, they should be considered exploratory and require replication in independent, multisite cohorts and across different sensor platforms to assess their generalizability and reproducibility. Third, potential confounding by demographic and disease-related factors, as well as dopaminergic treatment and treatment modality, cannot be excluded. In particular, the higher LEDD observed in the HMC cluster may reflect greater therapeutic requirements associated with motor complications but may also have influenced the wearable-derived motor profile. Finally, the digital profiles were derived exclusively from the motor features of a single waist-worn wearable device. Integrating additional sensors placed on other body segments, such as the upper limbs, may provide complementary information on motor performance and enable a more comprehensive multidimensional digital profiling approach.

## 5. Conclusions

This cross-sectional observational study provides the first evidence that unsupervised analysis of prolonged home-based wearable monitoring data in PD can identify distinct digital motor profiles. Our findings suggest that digital motor profiling can distinguish patients with a high versus low burden of motor complications despite similar disease duration and clinical stage, capturing a clinically relevant dimension of disease heterogeneity that is not reflected by clinical staging alone. Because motor complications fluctuate over time and are strongly influenced by medication cycles and environmental demands, continuous home-based monitoring provides an objective and ecologically valid tool to quantify their expression under real-world conditions. Among the digital features examined, dyskinesia-related measures appear to have the greatest potential for identifying patients with a higher burden of motor complications under real-world conditions. These exploratory findings may support future patient stratification but require prospective clinical validation in independent cohorts before their clinical application.

## Figures and Tables

**Table 1:** Demographic and clinical characteristics of study cohort.

Characteristics of Study Cohort (*n* = *76*)	Data
**Demographics**	
Age, years	68.68 ± 10.08
Sex, male/female, *n* (%)	55 (72.4)/21 (27.6)
Disease duration, years	9.20 ± 4.89
**Disease severity**	
Hoehn and Yahr (H&Y) stage	2.18 ± 0.52
Phenotype (A/T/M), *n* (%)	50 (65.8)/16 (21.1)/10 (13.2)
MDS-UPDRS Part I	10.92 ± 5.80
MDS-UPDRS Part II	12.11 ± 6.74
MDS-UPDRS Part III (ON)	21.93 ± 10.71
MDS-UPDRS Part IV	5.51 ± 4.48
Levodopa equivalent daily dose, mg/day	989.91 ± 483.66
**Motor complications**	
UDysRS Part III	3.07 ± 4.21
UDysRS Part IV	2.18 ± 3.00
Wearing-Off Questionnaire-19	4.00 ± 3.70
Freezing of Gait Questionnaire	4.50 ± 5.54
**Cognitive and neuropsychiatric assessment**	
Montreal Cognitive Assessment	24.30 ± 5.17
Frontal Assessment Battery	14.24 ± 3.31
Beck Depression Inventory-II	7.86 ± 6.04
**Patient-reported outcomes**	
Parkinson’s Disease Questionnaire-39	25.68 ± 14.74
Parkinson’s Disease Sleep Scale	70.14 ± 51.50

Data are presented as mean ± standard deviation (SD) or *n* (%), as appropriate. A, akinetic-rigid; T, tremor-dominant; M, mixed phenotype; MDS-UPDRS, Movement Disorder Society–Unified Parkinson’s Disease Rating Scale; UDysRS, Unified Dyskinesia Rating Scale.

**Table 2:** Clinical and demographic comparison between the identified clusters.

Characteristics	HMC (Cluster 0, *n* = 33)	LMC (Cluster 1, *n* = 43)	Effect Size	Adjusted *p*
**Demographics**				
Age, years	68.0 (59.0–74.0)	72.0 (65.0–77.5)	0.198	0.216
Disease duration, years	10.0 (7.0–12.0)	7.0 (4.5–12.0)	−0.309	0.052
Age at disease onset, years	55.3 ± 9.9	61.1 ± 10.6	0.563	0.052
Sex, male/female, *n* (%)	19 (57.6)/14 (42.4)	36 (83.7)/7 (16.3)	0.260	0.052
**Disease severity**				
Hoehn and Yahr	2.0 (2.0–3.0)	2.0 (2.0–2.0)	0.269	0.052
Phenotype (A/T/M), *n* (%)	24 (72.7)/6 (18.2)/3 (9.1)	26 (60.5)/10 (23.2)/7 (16.3)	0.135	0.624
MDS-UPDRS Part I	10.0 (8.0–14.0)	9.0 (5.5–15.0)	−0.072	0.696
MDS-UPDRS Part II	12.0 (8.0–17.0)	11.0 (6.5–15.5)	−0.176	0.271
MDS-UPDRS Part III (ON)	20.5 (14.0–25.0)	19.0 (17.0–28.5)	−0.043	0.836
Levodopa equivalent daily dose, mg/day	1160 (965–1464)	657 (500–1120)	−0.577	**<0.001**
**Motor complications**				
MDS-UPDRS Part IV	7.0 (4.0–12.0)	3.0 (0.0–5.5)	−0.546	**<0.001**
UDysRS Part III	3.0 (1.0–9.0)	0.0 (0.0–2.0)	−0.496	**<0.001**
UDysRS Part IV	2.0 (1.0–6.0)	0.0 (0.0–2.0)	−0.458	**0.002**
Wearing-Off Questionnaire−19	4.0 (2.0–8.0)	2.0 (0.0–5.0)	−0.385	**0.016**
Freezing of Gait Questionnaire	4.0 (0.0–11.0)	0.0 (0.0–7.5)	−0.214	0.173
**Cognitive and neuropsychiatric assessment**				
Montreal Cognitive Assessment	26.0 (23.0–28.0)	25.0 (23.0–26.5)	−0.211	0.194
Frontal Assessment Battery	15.0 (13.0–17.0)	15.0 (12.5–16.0)	−0.126	0.462
Beck Depression Inventory-II	5.0 (4.0–10.0)	7.0 (4.0–10.5)	0.034	0.847
**Patient-reported outcomes**				
Parkinson’s Disease Questionnaire−39	25.0 (15.0–33.0)	22.0 (14.5–36.5)	−0.004	0.979
Parkinson’s Disease Sleep Scale	75.0 (10.0–108.0)	92.0 (19.0–123.0)	0.225	0.173

Data are presented as mean ± standard deviation (SD), median (interquartile range [IQR]), or *n* (%), as appropriate. Significant adjusted *p* values are shown in bold. A, akinetic-rigid; HMC, High Motor Complication; LMC, Low Motor Complication; M, mixed phenotype; MDS-UPDRS, Movement Disorder Society–Unified Parkinson’s Disease Rating Scale; T, tremor-dominant; UDysRS, Unified Dyskinesia Rating Scale. Negative effect sizes indicate higher values in the HMC cluster, whereas positive effect sizes indicate higher values in the LMC cluster.

**Table 3:** Comparison of sensor-based measures between the identified clusters.

Variable	HMC (Cluster 0, *n* = 33)	LMC (Cluster 1, *n* = 43)	Effect Size	Adjusted *p*
Norm number of walking bouts	2.778 (2.087–3.703)	2.742 (1.789–4.427)	−0.033	0.883
Norm movement amount	0.446 ± 0.086	0.346 ± 0.077	−1.228	<0.001
Mean stride fluidity	8.558 ± 1.384	7.375 ± 1.060	−0.976	<0.001
Mean stride fluidity variability (CV, %)	1.933 (1.440–2.212)	1.292 (1.055–1.497)	−0.663	<0.001
Mean step length (m)	0.832 ± 0.123	0.832 ± 0.121	0.002	0.992
Mean stride speed (m/s)	0.537 ± 0.088	0.572 ± 0.099	0.368	0.133
Mean cadence (steps/min)	38.876 ± 2.631	40.984 ± 2.796	0.773	0.002
Norm number of FOG episodes	0.006 (0.003–0.009)	0.001 (0.001–0.005)	−0.430	0.002
Norm FOG duration	0.006 (0.003–0.010)	0.002 (0.001–0.006)	−0.409	0.003
Norm number of postural transitions	0.851 (0.649–1.064)	0.361 (0.211–0.544)	−0.805	<0.001
Mean Dyskinesia Power	0.590 (0.488–0.743)	0.351 (0.295–0.405)	−0.917	<0.001
Norm dyskinesia	0.082 (0.059–0.127)	0.023 (0.009–0.048)	−0.762	<0.001

FOG, freezing of gait. Norm denotes variables expressed per unit of valid monitoring time (wear time), thereby accounting for differences in recording duration across participants. Mean Dyskinesia Power: average dyskinesia intensity; Norm dyskinesia: normalized dyskinesia duration. Negative effect sizes indicate higher values in the HMC cluster, whereas positive effect sizes indicate higher values in the LMC cluster.

**Table 4:** Classification performance of the random forest surrogate model.

Fold	Accuracy	Balanced Accuracy	F1-Score
1	0.875	0.857	0.90
2	1.00	1.00	1.00
3	0.867	0.888	0.875
4	1.00	1.00	1.00
5	0.933	0.928	0.941
Mean ± SD	0.935 ± 0.065	0.935 ± 0.065	0.943 ± 0.057

## Data Availability

The dataset used in this study can be obtained from the corresponding author upon reasonable request. Access to sensitive data will be restricted to preserve privacy.

## Supplementary Materials

The following supporting information can be downloaded at https://www.mdpi.com/article/10.3390/brainsci16101074/s1, Supplementary Material S1: “STROBE checklist”; Supplementary Material S2: “Clustering procedures and sensitivity analyses”.

## Abbreviations

The following abbreviations are used in this manuscript:

ARI	Adjusted Rand Index
BDI-II	Beck Depression Inventory-II
CH	Calinski–Harabasz
CV	Coefficient of variation
FAB	Frontal Assessment Battery
FDR	False discovery rate
FOG	Freezing of gait
FOG-Q	Freezing of Gait Questionnaire
H&Y	Hoehn and Yahr
HMC	High Motor Complication
IQR	Interquartile range
KNN	K-nearest neighbors
LEDD	Levodopa equivalent daily dose
LMC	Low Motor Complication
MDS-UPDRS	Movement Disorder Society–Unified Parkinson’s Disease Rating Scale
MoCA	Montreal Cognitive Assessment
PCA	Principal component analysis
PD	Parkinson’s disease
PDQ-39	Parkinson’s Disease Questionnaire-39
PDSS	Parkinson’s Disease Sleep Scale
SHAP	SHapley Additive exPlanations
STROBE	Strengthening the Reporting of Observational Studies in Epidemiology
UDysRS	Unified Dyskinesia Rating Scale
WOQ-19	Wearing-Off Questionnaire-19
