# Supplementary material for: Digital Motor Profiling in Parkinson’s Disease: Long-Term Monitoring Through Wearable Sensors
Source: Brain Sci. 2026 Oct 7;16(10):1074. doi: 10.3390/brainsci16101074 (PMC13702610; doi:10.3390/brainsci16101074)
Supplement: Supplementary file 1 [file brainsci-16-01074-s001.zip › Supplementary Material S2_Clustering procedures and sensitivity analyses.pdf]

Supplementary Materials and Methods

Clustering procedures

Unsupervised clustering was performed using the K-means algorithm applied to the PCA-transformed feature space. The optimal number of clusters was evaluated over a range of candidate values from 2 to 14 clusters. Cluster quality was assessed using multiple internal validation metrics: Silhouette score, measuring within-cluster cohesion and between-cluster separation, and Calinski–Harabasz index, evaluating the ratio between inter-cluster dispersion and intra-cluster variance. The optimal number of clusters was automatically selected by combining normalized Silhouette and Calinski–Harabasz scores into a composite clustering quality score. The value of k maximizing this combined score was selected for the final clustering model. Silhouette score and Calinski–Harabasz index were calculated for different values of k.

For each subject i, the silhouette coefficient is:

$$s(i) = \frac{b(i) - a(i)}{\max(a(i)b(i))}$$

Here:

- $a(i)$= average distance between subject i and all other subjects within the same cluster (within-cluster cohesion)

$$a(i) = \frac{1}{|C_i| - 1} \sum_{j \in C_i, j \neq i} d(i, j)$$

- $b(i)$= minimum average distance between subject i and subjects belonging to another cluster (nearest-cluster separation)

$$b(i) = \min_{k \neq C_i} \left(\frac{1}{|C_k|} \sum_{j \in C_k} d(i, j) \right)$$

The overall silhouette score was obtained as the average value across all subjects:

$$S = \frac{1}{n} \sum_{i=1}^{n} s(i)$$

Here, n is the number of subjects. Higher S indicates that subjects are well matched to their assigned cluster and separated from other clusters.

The Calinski–Harabasz index is defined as:

$$CH(k) = \frac{\mathrm{Tr}(B_k)/(k-1)}{\mathrm{Tr}(W_k)/(n-k)}$$

Here:

- k= number of clusters;
- n= number of subjects;
- B_k= between-cluster dispersion matrix;
- W_k= within-cluster dispersion matrix;
- Tr= sum of diagonal elements.

The within-cluster dispersion is:

$$W_k = \sum_{q=1}^{k} \sum_{x_i \in C_q} (x_i - \mu_q)(x_i - \mu_q)^T$$

Here:

- C_q= cluster q;
- μ_q= centroid of cluster q.

The between-cluster dispersion is:

$$B_k = \sum_{q=1}^{k} n_q (\mu_q - \mu)(\mu_q - \mu)^T$$

Here:

- n_q= number of subjects in cluster q;
- μ= global centroid of the dataset.

Higher CH values indicate clusters that are internally compact and externally well separated. After calculating both indices for each candidate number of clusters k, they were normalized using min–max scaling:

$$S_{norm}(k) = \frac{S(k) - S_{min}}{S_{max} - S_{min}}$$

$$CH_{norm}(k) = \frac{CH(k) - CH_{min}}{CH_{max} - CH_{min}}$$

Then, the composite score was defined as:

$$Composite(k) = \frac{S_{norm}(k) + CH_{norm}(k)}{2}$$

Finally, the selected number of clusters was obtained as:

$$k^* = \arg\max_k Composite(k)$$

A final K-means model was fitted using the selected number of clusters, with 100 random initializations to improve centroid stability. Cluster assignments generated by this model were used for subsequent analyses.

Cluster robustness was evaluated using bootstrap resampling. A reference K-means clustering solution was first obtained from the complete dataset. Subsequently, 200 bootstrap datasets were generated by sampling observations with replacement. For each bootstrap iteration, K-means clustering was independently performed using the selected number of clusters. The similarity between bootstrap cluster assignments and the reference clustering was quantified using the Adjusted Rand Index (ARI):

$$ARI = \frac{\sum_{ij} \binom{n_{ij}}{2} - \frac{\left(\sum_i \binom{a_i}{2}\right)\left(\sum_j \binom{b_j}{2}\right)}{\binom{n}{2}}}{\frac{1}{2}\left[\sum_i \binom{q}{2} + \sum_j \binom{b_j}{2}\right] - \frac{\left(\sum_i \binom{a_i}{2}\right)\left(\sum_j \binom{b_j}{2}\right)}{\binom{n}{2}}}$$

Here:

- n= total number of observations;
- a_i= size of cluster i in the first partition;
- b_j= size of cluster j in the second partition;

- n_{ij}= overlap between cluster i and cluster j.

Mean, standard deviation, minimum, and maximum ARI values were calculated to evaluate clustering stability.

Sensitivity analysis

To assess the robustness of the clustering solution to the representation of the input data and to the contribution of specific sensor-derived motor domains, three complementary sensitivity analyses were performed. Each analysis otherwise followed the same preprocessing, cluster-selection, and clustering framework as the primary analysis. The primary clustering solution based on the complete feature set was used as the reference partition for comparisons.

The robustness of the clustering solution to dimensionality reduction was evaluated by comparing clustering performed in PC space with clustering performed directly on the complete standardized feature set. In the PCA-based analysis, PCA was applied after standardization, and the minimum number of components explaining at least 90% of the total variance was retained. K-means clustering was then performed in the retained PC space. In the full-feature analysis, K-means clustering was instead performed directly on the complete standardized feature matrix, without PCA. The same candidate range of cluster numbers was evaluated in both analyses, and the optimal number of clusters was determined using the same combined internal validation criterion based on the Silhouette coefficient and Calinski–Harabasz index. The resulting partitions were compared using the ARI, NMI, and cluster-level agreement. The latter was assessed after optimal matching of cluster labels using the Hungarian algorithm and was expressed as the proportion of subjects assigned to the same matched cluster, together with differences in cluster size between the two solutions.

The original 30 features used as input data were a priori assigned to five sensor-derived motor domains according to their clinical and functional meaning. The *Gait* domain comprised the mean and coefficient-of-variation (CV) measures of stride fluidity, filtered stride fluidity, stride fluidity variability, step length, stride speed, and cadence (12 features). The *Dyskinesia* domain comprised mean dyskinesia power, mean dyskinesia continuity, CV dyskinesia power, CV dyskinesia continuity, and the dyskinesia motor-state variable (5 features). The *FOG* domain comprised the normalized number of FOG episodes and normalized FOG duration (2 features). The *Mobility* domain comprised the normalized number of walking bouts, movement amount, number of steps, time walking, time lying, number of postural transitions, and number of falls (7 features). The *Motor state* domain comprised the normalized time in OFF condition, ON condition, bradykinesia in the OFF condition, and bradykinesia in the ON condition (4 features).

The contribution of individual sensor-derived motor domains to the overall clustering structure was assessed using a leave-one-domain-out approach. Five separate analyses were performed, each excluding all features belonging to one predefined domain while retaining the remaining features. Thus, clustering was repeated after excluding the *Gait, Dyskinesia, FOG, Mobility,* and *Motor state* domains. For each reduced feature set, the complete preprocessing and clustering procedure was repeated de novo, including standardization, PCA,

determination of the optimal number of clusters, and K-means clustering. Each resulting partition was compared with the primary clustering solution based on the complete feature set using subject-level cluster agreement. This analysis was intended to determine whether the identified clustering structure was dependent on any single sensor-derived motor domain.

Finally, the ability of individual sensor-derived motor domains to independently reproduce the multivariate clustering structure was assessed using domain-only analyses. Separate clustering analyses were performed using exclusively the features belonging to each of the five predefined domains: *Gait, Dyskinesia, FOG, Mobility,* and *Motor state*. For each domain, the corresponding feature subset was analyzed independently using the same preprocessing and clustering framework as the primary analysis. The optimal number of clusters and internal clustering metrics were recorded, and the resulting partitions were compared with the primary clustering solution using subject-level cluster agreement after optimal cluster-label matching.

Supplementary Results

Sensitivity analysis

The comparison between the PCA-based (11 components, 91.6% explained variance) and full-feature (30 features) clustering solutions showed complete agreement in the original dataset (100% subject-level agreement; ARI = 1.00; NMI = 1.00). Both approaches selected the same number of clusters (k = 2) and showed similar clustering quality. **Figure S1** shows the Silhouette score (left) and composite score (right) across the two feature representations.

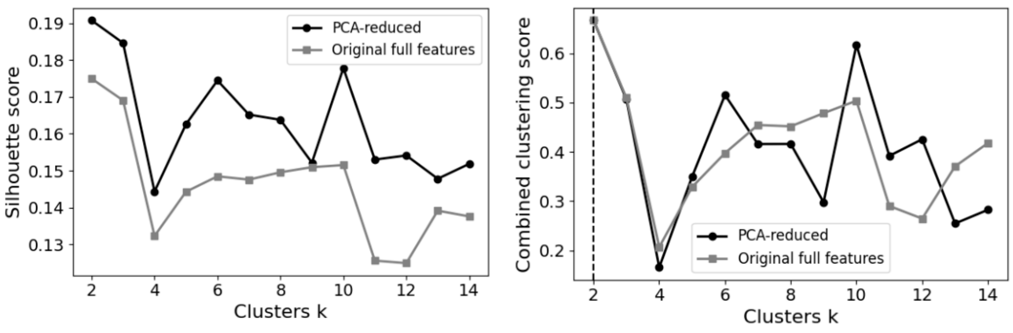

Figure S1. Comparison of PCA-based (11 components) and full feature set (30 features) clustering quality. Silhouette score (left) and composite score (right) are compared across candidate clustering solutions (k = 2–14).

Across bootstrap iterations, the PCA-based and full-feature approaches showed comparable stability (PCA: ARI = 0.80 ± 0.18, NMI = 0.76 ± 0.17; Full: ARI = 0.80 ± 0.18, NMI = 0.76 ± 0.17), while agreement between the two clustering solutions remained very high (ARI = 0.98 ± 0.06; NMI = 0.96 ± 0.07). Accordingly, the dimensionality reduction did not substantially affect the identified clustering structure (**Table S1**).

Table S1. Comparison of cluster stability across PCA-reduced and Full feature set.

Feature set	ARI (mean ± SD, min–max)	NMI (mean ± SD, min–max)
PCA	0.80 ± 0.18 (0.10-1.0)	0.76 ± 0.17 (0.16-1.0)
Full	0.80 ± 0.18 (0.10-1.0)	0.76 ± 0.17 (0.16-1.0)
PCA vs Full	0.98 ± 0.06 (0.71-1.0)	0.96 ± 0.07 (0.69-1.0)

The contribution of individual sensor-derived motor domains was assessed by comparing the effects of their exclusion or individual inclusion with the original feature set (**Figure S2**). Excluding dyskinesia resulted in the greatest reduction in performance, with subject-level cluster agreement decreasing to 65.8%. In contrast, excluding gait, FOG, motor state, or mobility resulted in substantially smaller changes, with relative performance remaining above 96%. When each single domain was used as the input data, performance was highest for dyskinesia (90.8%), followed by motor state (69.7%), mobility (67.1%), gait (63.2%), and FOG (59.2%).

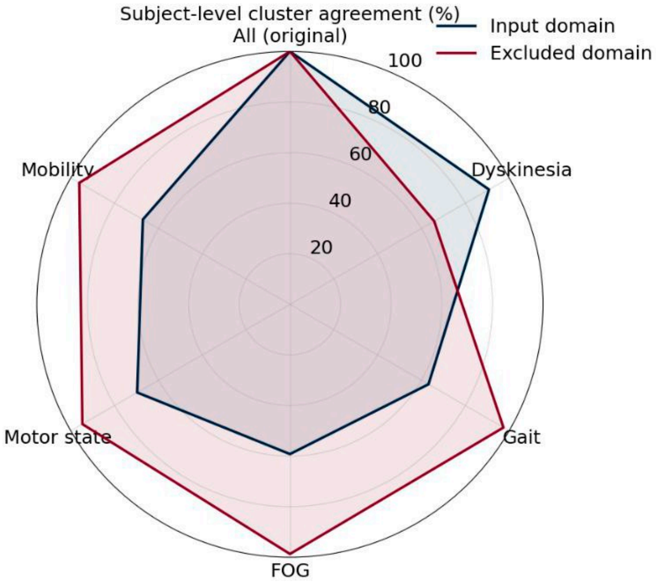

Figure S2. Subject-level clustering agreement across different input feature domains. The blue line shows the results when a single domain is used as input data. The orange line shows the results when each individual domain is excluded from the input data.

Despite the prominent contribution of sensor-derived dyskinesia features to the clustering structure, cluster membership did not simply correspond to the presence or absence of clinically assessed dyskinesia. Indeed, in the HMC cluster, 28 of 33 patients (85%) had clinically detectable dyskinesia (UDysRS-III > 0), whereas in the LMC cluster, 27 of 43 patients (63%) had no clinically detectable dyskinesia (UDysRS-III = 0).
